# Analytical validation of a hybrid-approach combining tumor-informed and tumor-agnostic bespoke ctDNA panel assay for the sensitive detection of minimal residual disease

**DOI:** 10.1371/journal.pone.0334282

**Published:** 2025-11-10

**Authors:** Sunghoon Heo, Seon-Kyu Ham, Hayoon Lee, Bom Han, Hanseong Roh, Seongmun Jeong, Hwang-Phill Kim, Duhee Bang, Sang-Hyun Song, Tae-You Kim

**Affiliations:** 1 IMBdx, Inc., Seoul, Korea; 2 Department of Chemistry, Yonsei University, Seoul, Korea; 3 Department of Internal Medicine, Seoul National University Hospital, Seoul, Korea; 4 Cancer Research Institute, Seoul National University, Seoul, Korea; 5 Department of Molecular Medicine and Biopharmaceutical Sciences, Graduate School of Convergence Science and Technology, Seoul National University, Seoul, Korea; University of Maryland College Park, UNITED STATES OF AMERICA

## Abstract

Minimal residual disease (MRD) is a small group of cancer cells not eliminated by anti-cancer treatment. Because of its small size, conventional imaging system may not be able to detect the MRD in routine clinical practice. Although the liquid biopsy tests can detect the circulating tumor DNA (ctDNA) when the tumor is present in the body, the fraction of ctDNA is considered lower than the 0.01% which is unreachable by current state-of-the-art liquid biopsy assay relying on fixed-gene panel approach. Here, we describe the analytical validation result of our previously developed a tumor-informed MRD test, CancerDetect^TM^ (formerly reported as AlphaLiquid^®^Detect), leveraging large-scale mutation spectrum profiling strategy to enhance detection sensitivity. The CancerDetect^TM^ is a hybrid-approach MRD test combining both personalized (bespoke) mutations and tumor-agnostic clinically actionable targets (hotspot mutations) with hybridization capture technology. The analytical validation result of CancerDetect^TM^ showed limit of detection successfully reached down to 0.001% (10^−5^) with 99.9% specificity.

## Introduction

Recent advancements in liquid biopsy technologies have conferred upon medical oncologists an unparalleled ability to detect previously undetectable neoplasms, with particular emphasis on minimal residual disease (MRD). MRD represents a well-characterized harbinger of cancer recurrence, frequently evading detection by conventional clinical methodologies due to its minuscule size [1]. The underlying principle of liquid biopsy in oncology posits that apoptotic or necrotic cancer cells release their DNA into the circulatory system, referred to as circulating tumor DNA (ctDNA). This makes liquid biopsy a promising modality for MRD detection, even in scenarios where the neoplastic burden is exceedingly low. However, the concentration of ctDNA in a patient’s circulatory system is typically less than 0.01%, thereby necessitating ultra-sensitive detection methodologies [2]_._ Further complicating this endeavor is the intrinsic error rate of modern sequencing technologies, which approximates 0.1%, rendering it challenging to discern authentic oncogenic mutations from sequencing artifacts [3–5].

In the development of MRD detection technologies, two primary paradigms have emerged: tumor-agnostic approaches, which eschew reliance on tumor-specific information, and tumor-informed approaches, which are predicated on such information. Advocates of each paradigm have demonstrated the clinical utility of MRD detection, particularly in stratifying the risk of cancer relapse and in the longitudinal surveillance of therapeutic efficacy post-surgery.

Tumor-agnostic approaches leverage pre-configured panels encompassing dozens to hundreds of genes implicated in oncogenesis. These methodologies typically employ unique molecular identifiers (UMIs) incorporated into sequencing adapters to mitigate sequencing errors, combined with deep sequencing [6,7]. Clinical studies utilizing tumor-agnostic strategies have demonstrated that incomplete clearance of ctDNA is correlated with adverse patient outcomes [8–10]. While these methods have illustrated the practical utility of tumor-agnostic approaches for MRD detection in a streamlined manner, the detection limit, which reaches approximately 0.1%, remains suboptimal for the sensitive detection of MRD [11].

Tumor-informed approaches, on the other hand, employ custom-designed target panels tailored to detect specific cancer mutations identified from the patient’s tumor tissue specimen. These methodologies typically select fewer than 50 mutations and utilize polymerase chain reaction (PCR) to amplify cell-free DNA (cfDNA) samples extracted from the patient, thereby increasing the detection sensitivity for ctDNA across the targets of interest [12–14]. With this tumor-informed strategy, the detection threshold is reduced to approximately 0.01%. Owing to this heightened sensitivity compared to tumor-agnostic approaches, various clinical trials have underscored the utility of tumor-informed strategies, including the identification of patients at high risk of recurrence post-surgery and the monitoring of treatment efficacy following adjuvant chemotherapy or immunotherapy [15–19]. Despite the enhanced detection sensitivity afforded by tumor-informed technologies, their performance remains insufficient for the ultra-sensitive detection of MRD.

Given the trace amounts of ctDNA, enhancing the likelihood of ctDNA detection is paramount for improving detection sensitivity. Three strategies are available to achieve this objective: increasing the total DNA input, augmenting sequencing depth, and expanding the mutational search space. The first strategy is constrained by practical limitations in clinical settings, as the amount of DNA is directly proportional to the volume of blood drawn from the patient. While increasing sequencing depth may theoretically facilitate the detection of minute ctDNA levels, the associated costs are economically prohibitive, even considering recent reductions in sequencing costs. The remaining strategy, expanding the mutational search space, is clinically viable, as it can lower the required DNA input for MRD testing, thereby obviating the need for large volumes of blood. Simulations have indicated that a large-scale mutation profiling strategy, combined with moderate sequencing depth, could achieve a detection limit below 0.01% [20,21]. Additionally, we have previously shown that the strategy is effective for not only lowering the detection limit but also increases the clinical sensitivity [22]. Consequently, expanding the ctDNA search space represents a pragmatic approach for achieving sensitive MRD detection.

In this context, we present the analytical performance of CancerDetect^TM^, formerly known as AlphaLiquid^®^Detect, a tumor-informed, personalized panel assay that adopts a large-scale mutation profiling strategy. Differ from the previous version, we applied a hybrid-approach combining both tumor-informed and tumor-agnostic strategy. The CancerDetect^TM^ assay is capable of achieving a detection limit as low as 0.001%, thereby setting a new benchmark for the sensitivity of MRD detection.

## Materials and methods

### Sample preparation and sequencing

#### Reference material sample preparation for limit of detection analysis.

To determine the minimum amount of input DNA required to maintain the LoD in our experiment, we mixed the well-characterized cell lines NA12891 and NA12892 (Coriell Institute, USA) to generate various VAFs. The gDNA extracted from these cell lines was quantified using the Qubit dsDNA High Sensitivity Kit (Thermo Fisher Scientific, USA) and then fragmented to a target size of 180 bp, similar to typical cell-free DNA, using a Covaris S220 sonicator (Covaris Inc., USA). The sheared gDNA was quantified using the D1000 ScreenTape assay (Agilent Technologies, USA), and the sheared NA12891 gDNA was mixed with sheared NA12892 gDNA at mass ratios of 0.5%, 0.1%, 0.05%, 0.01%, 0.005%, 0.001%, and 0.0001%. These mixtures were quantified using the D1000 ScreenTape assay and used to create index-tagged libraries to determine detection limits.

Additionally, to assess the detection limits for hotspot mutations frequently observed in cancer, we conducted experiments using the Seraseq^®^ ctDNA MRD Panel Mix (LGC SeraCare, USA) at VAFs of 0.5%, 0.1%, 0.05%, 0.01%, 0.005%, 0.001%, and 0%. The DNA was quantified using the cfDNA Tapestation Assay (Agilent Technology, USA), and VAFs not included in the commercial kit were prepared by diluting the provided 0% with the desired mass ratios. The ctDNA was used to create index-tagged libraries with a maximum input amount of 30 ng, as specified by the CancerDetect™ assay.

#### Reference material sample preparation for precision and reproducibility.

Thirty nanograms of 0.001% sheared gDNA mixture and blank samples were processed by three different operators using three different instruments over three days by varying the lot number of hybridization capture kit (Twist Bioscience, USA).

#### Reference material sample preparation for the matrix interference test.

To determine whether components from the blood or DNA extraction kit could cause interference and affect the analysis during the CancerDetect™ experiment, an interference test was conducted. For this test, 120 ng each of reference materials, Seraseq® ctDNA MRD Panel Mix VAF 0.01% and WT, were added to 4 ml of pooled human plasma (Innovative Research, USA) to accurately compare MRD positive/negative status. Also, to assess the precision of the LoD_95,_ 120 ng each of the sheared gDNA mixture VAF 0.001% and sheared NA12891 gDNA, were added to 4 ml of plasma. The selected interfering substances were endogenous materials (Bilirubin, Hemoglobin) and exogenous materials (Wash buffer, EDTA), which could potentially be introduced during the experimental process. The control group did not receive any interfering substances. For interference test, bilirubin (Molecular Depot, USA) and hemoglobin (Molecular Depot, USA) were added to the plasma at 20 mg/dL and 5 mg/mL before DNA extraction. For the exogeneous material test ETDA (Sigma, USA) and wash buffer (QIAGEN, USA) was added to the final elution buffer at 20% after extraction [23–25]. All plasma samples were centrifuged at 3,134 g for 10 minutes according to IMBdx standard operating procedure, and the supernatant, excluding cell debris, was collected. ctDNA was extracted using the Maxwell^®^ RSC ccfDNA Plasma Kit (Promega Corporation, USA). The extracted DNA was quantified using cell-free DNA ScreenTape analysis (Agilent Technologies, USA), and libraries were prepared in triplicate with 30 ng of DNA each.

#### Library preparation and DNA sequencing.

Index-tagged libraries were generated with 3–20 replicates for each input and variant allele frequency following the CancerDetect™ protocol. These libraries were then quantified using the D1000 ScreenTape assay (Agilent, USA). Up to 1000 ng of index-tagged library DNA was hybridized and captured using a BSP (Twist Bioscience, USA) designed to target the selected mutations, followed by enrichment. The final target-captured libraries were quantified using the D1000 ScreenTape assay. Each target-captured library was diluted to 2 nM, pooled, and then quantified using both the High Sensitivity D1000 ScreenTape assay (Agilent, USA) and a Commercial Library Quantification kit (KAPA, USA). Sequencing was performed on the Illumina NovaSeq 6000 platform (Illumina, USA) using 2x150 bp paired-end reads, aiming for an average on-target coverage of 100,000x.

### Data analysis

#### Design of bespoke panel.

The germline variants of two cell lines were compared and selected according to their genotype. Secondly, we filtered out targets those did not pass the criteria of the CancerDetect^TM^ variant selection algorithm. Among filtered genotypes, we selected genotypes uniformly distributed across chromosome considering the size. Total 385 SNPs were selected to design the panel. Fifty-eight clinically actionable target regions were also included with these selected SNPs in the panel.

#### Data preprocessing.

The sequencing data was processed as previously described^20^. In brief, the FASTQ files were trimmed using fastp software and UMI was extracted. Reads were aligned onto human reference genome (hg38) using bwa [26]. Consensus calls were generated using fgbio software and applied option was differ by the target region of interest [27]. BAM file consist of consensus reads were re-aligned onto reference genome and quality filter was applied. The resulting BAM file was used to call variants.

#### Variant discovery and MRD Detection.

From the BAM file, variant calls were performed using VarDict software and further in-house script was used to filter variants [28]. The proprietary HQS^TM^ technology was applied to remove false positives generated from the sequencing errors. Hard filters including base quality, read position, mapping quality was applied to the final variant call set. We excluded targets ambiguous to get the mixed fraction. After loci filtration, variant allele frequency was calculated to estimate the observed variant allele frequency. Resulting variants were used to determine the MRD status. Sample was MRD positive if the number of mutations detected were greater than or equal to 2 [22].

#### Statistical analysis.

All statistical analysis was performed using R software (version 4.1.1). The p-value was tested using two-sided Student’s t-test unless specified.

## Results

### CancerDetect^TM^: A hybrid approach combining tumor-informed and tumor-agnostic strategy utilizing targeted capture sequencing technology

The previous iteration of CancerDetect^TM^ was a tumor-informed MRD test employing a large-scale mutation profiling strategy [22]. While tumor-informed tests enhance detection sensitivity, they are inherently limited in their ability to identify *de novo* mutations arising during the course of cancer treatment. To address this limitation, the CancerDetect™ panel has been augmented to integrate regions targeting 58 clinically actionable mutations across 15 genes (S1 Table) into the panel. Finally, the bespoke panel (BSP) is consist of tumor-informed private mutation target regions (bespoke regions) and tumor-agnostic mutation target regions (hotspot regions).

**Fig 1:**
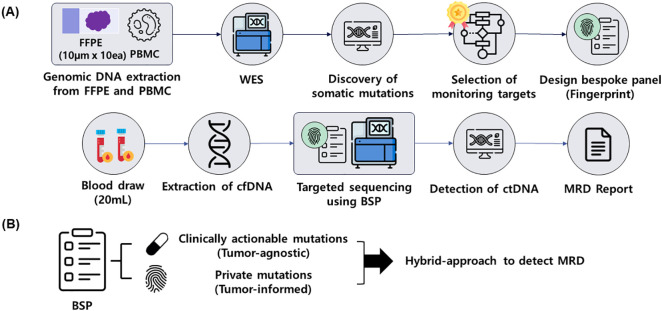
The procedure of CancerDetect^TM^. **(A)** The procedure of CancerDetect^TM^ starts from comprehensive exome-wide sequencing to design bespoke panel (BSP) using a proprietary selection algorithm. The designed BSP was used to detect ctDNA from the patient. **(B)** The BSP of CancerDetect^TM^ consist of patient’s personal target variants and the clinically actionable variants across 15 genes.

The entire workflow of CancerDetect^TM^ has been revised from previous versions. The process begins with comprehensive exome-wide profiling to identify somatic mutations in the patient’s tumor specimen and matched normal tissue. A subset of these somatic mutations is then selected using a proprietary variant selection algorithm, which informs the design of the BSP. Targeted capture sequencing is subsequently performed on the patient’s cell-free DNA (cfDNA) specimen using the BSP, and the data is analyzed with the proprietary HQS^TM^ technology to minimize sequencing errors. The integration of results from both the bespoke and hotspot regions ultimately determines the patient’s current MRD status (Fig 1).

### CancerDetect^TM^ can detect as low as 0.001% of ctDNA

**Fig 2:**
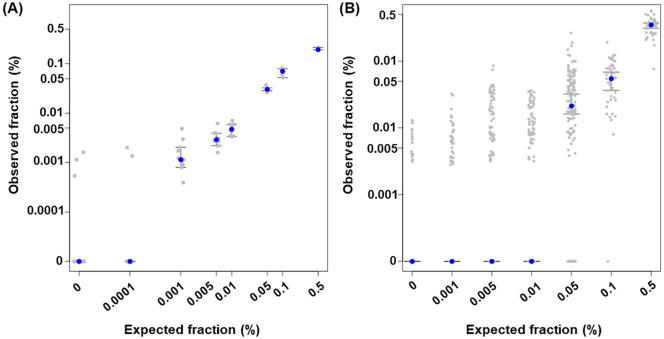
The limit of detection (LoD) of CancerDetect^TM^. **(A)** The LoD test result of bespoke regions and (B) test result of hotspot regions. Blue dots indicate the median observed fraction. Gray dots indicate the observed fraction of each sample (A) or the variant itself **(B)**. Error bars indicate the 95% confidence interval considering median.

The limit of detection (LoD_95_) infers that the quantifiable amount of analyte across 95% of samples. Since the target composition of BSP is bespoke mutation and *de novo* mutation of which the objective is different, measuring the LoD_95_ requires different approach for each target region type. To accomplish this, we generated two types of reference material using germline known cell lines and commercially available reference standards. For private mutation target regions (bespoke regions), the cell line mixture of known germline mutation was used to determine the LoD_95_. The commercial reference standard was used to measure the LoD_95_ of *de novo* mutation target regions (hotspot regions). By varying the ratio, the LoD_95_ of bespoke regions were determined as 0.001% and the LoD_95_ of hotspot regions was determined as 0.1% (Fig 2)

### CancerDetect^TM^ can discriminate signals of blank samples with high specificity

The specificity was defined as the proportion of false positive variants observed across target loci in the wild-type reference materials. For bespoke regions, twenty wild type cell line reference materials were used, and overall false positive rate was 0.3% on average resulted in 99.9% specificity. For hotspot regions, analytical specificity was evaluated for the targets covered by the panel. Total twenty commercial 0% reference materials were used and 95% of analytical specificity was observed (Table 1).

**Table 1:** The key performance summary of CancerDetect^TM.^

Metric	Bespoke region (95% CI)	Hotspot region (95% CI)
Limit of detection	0.001%	0.1%
Limit of blank	0.0003%	0.003%
Specificity	99.9% (99.4-99.9)	95.0% (88.7-98.4)
Sensitivity	100% (96.4-100)	95.0% (88.7-98.4)

The limit of blank (LoB) refers to the highest analyte (DNA) concentration expected to be found from the wild type sample. It is known that the LoB could be calculated from the mean and standard deviation of the observed signal in blank samples [29], the LoB of CancerDetect^TM^ was determined as 0.0003% for bespoke region and 0.003% for hotspot region (Table 1).

### Precision of the CancerDetect^TM^ text

To test the precision of CancerDetect^TM^ test, we analyzed 18 samples of 0.001% and blank samples and measured the precision using coefficient of variation (CV) metric. As expected, higher CV was observed (i.e., sensitive to outlier) in blank samples compared to the 0.001% samples (Table 2).

**Table 2:** The precision of CancerDetect^TM^ test. CV: Coefficient of Variation.

Sample	Tested samples	Mean observed fraction (%)	Standard deviation	CV (%)
0.001%	18	0.0009	0.0008	0.935
0%	18	0.0003	0.0007	2.39

### Repeatability of the CancerDetect^TM^ test

To test the repeatability of CancerDetect^TM^ test, we analyzed triplicates of 0.001% and blank samples with different reagent kit lot. For 0.001% samples analyzed, the median of observed ctDNA fraction was not differ by reagent kit and the MRD positivity was observed as expected. For blank samples, the median observed ctDNA fraction was below the limit of blank except one sample. However, this sample did not satisfy the MRD criteria of the test indicating that 100% samples are MRD negative as expected (Table 3).

**Table 3:** The repeatability of the CancerDetect^TM^ test.

	MRD positivity		Median observed ctDNA fraction (range, %)	
0.001%	Blank	0.001%	Blank
Lot1	100% (3/3)	0% (0/3)	0.0008 (0.0004-0.0016)	0 (0−0)
Lot2	66.7% (2/3)	0% (0/3)	0.0009 (0-0.0017)	0 (0-0.0009)

### Reproducibility of the CancerDetect^TM^ test

To test the reproducibility, we analyzed the triplicates of the 0.001% and blank samples with three different operators. For 0.001% samples analyzed, the median observed ctDNA fraction was not differ by operators. For blanks samples, the median observed ctDNA fraction was below limit of detection except one operator. However the samples did not satisfy MRD criteria of the test indicating that 100% samples are MRD negative as expected (Table 4).

**Table 4:** The reproducibility of the CancerDetect^TM^ test.

Operator	MRD positivity		Median observed ctDNA fraction (range, %)	
0.001%	Blank	0.001%	Blank
Operator 1	100% (3/3)	0% (0/3)	0.001 (0.0002-0.002)	0.001 (0.0002-0.0029)
Operator 2	66.7% (2/3)	33.3% (1/3)	0.0007 (0-0.002)	0 (0-0.0003)
Operator 3	100% (3/3)	0% (0/3)	0.0008 (0.0004-0.001)	0 (0−0)

### CancerDetect^TM^ result is not affected by the interfering matrix materials

To test whether the performance was hindered by the external matrix materials (i.e., interference mataerial), we selected bilirubin and wash buffer of enrichment reagent as test interfering materials. Because the BSP of CancerDetect^TM^ consist of hotspot and bespoke regions, we tested both regions differently using gDNA mixture samples and commercial reference standards (see Materials and methods). For bespoke regions, although wash buffer showed increased observed ctDNA fraction in wild type test all result showed there was no statistically significant difference between control and interference material (Table 5, S1 Fig). For hotspot regions, there was not statistically significant difference between control and interference material regardless of wash buffer test showed decreased sensitivity indicating the false positives (Table 6, S2 Fig). This result indicates that the result of CancerDetect^TM^ test is not affected by the interference material.

**Table 5:** Inteference material test of bespoke region of CancerDetect^TM^. P-value was tested using Wilcoxon’s rank sum test.

Sample	Target ctDNA fraction (%)	Observed ctDNA fraction (median, %)	p-value
No interference material	0	0	Control
Bilirubin	0	0	0.33
Wash buffer	0	0.0028	0.076
Hemoglobin	0	0.0009	0.54
EDTA	0	0.0007	0.68
No interference material	0.001	0.0011	Control
Bilirubin	0.001	0.0013	0.64
Wash buffer	0.001	0.0012	0.21
Hemoglobin	0.001	0.0014	0.64
EDTA	0.001	0	0.094

**Table 6:** Inteference material test of hotspot region of CancerDetect^TM^. P-value was tested using Wilcoxon’s rank sum test. The performance metric indicates specificity and sensitivity for 0% and 0.1% VAF samples respectively.

Sample	Target ctDNA fraction (%)	Sensitivity or Specificity	p-value
No interference material	0	100	Control
Bilirubin	0	100	1
Wash buffer	0	93.3	0.46
Hemoglobin	0	100	1
EDTA	0	93.3	0.31
No interference material	0.1	93.3	Control
Bilirubin	0.1	100	0.33
Wash buffer	0.1	93.3	0.68
Hemoglobin	0.1	100	0.12
EDTA	0.1	93.3	0.68

## Discussion

In this study, we evaluated the analytical performance of CancerDetect^TM^, a novel hybrid approach that integrates both tumor-informed and tumor-agnostic methodologies. This combined strategy facilitates sensitive detection of MRD by leveraging tumor-informed techniques while not overlooking de novo mutations in cancer patients. Moreover, the target capture sequencing method employed allows for extensive mutation profiling, thereby enhancing detection sensitivity.

We assessed key performance parameters, including the limit of detection (LoD_95_), analytical specificity, limit of blank (LoB), as well as repeatability and reproducibility under varying conditions. The limit of detection of CancerDetect^TM^ was determined to be 0.001%, which is within an acceptable range for MRD detection. At this lower detection limit, it is crucial to differentiate between false positives and true mutations to minimize false-positive results. Using the proprietary HQS^TM^ technology, the assay’s analytical specificity was determined to be 99.9%, indicating a high probability that the detected mutations originated from cancer cells. The estimated LoB was 0.0003%, and the test results demonstrated reproducibility across different operators and reagents under consistent conditions.

Because the variant discovery from the hotspot region was performed without prior knowledge of variant information to mimic the real-world tumor-agnostic ctDNA detection scenario, the LoD_95_ and LoB of hotspot regions was defined as 0.1% and 0.003% respectively which is higher than the bespoke region.

However, interference testing revealed false positives associated with the wash buffer. Our data demonstrated that incomplete removal of wash buffer during library preparation could affect test results, as evidenced by an increased false-positive rate when an excess amount of wash buffer was added to the test solution. Despite these occurrences, the CancerDetect^TM^ results did not show a statistically significant difference compared to control experiments, suggesting that meticulous wash buffer removal is necessary to ensure accurate test outcomes.

A notable limitation of the earlier version of CancerDetect^TM^ was its extended turnaround time (TAT) of 8–10 weeks. The revised version has successfully reduced the TAT to 4–6 weeks, making it more suitable for clinical applications such as deciding on adjuvant chemotherapy, which typically occurs within 4–8 weeks post-surgery.

While the TAT has been reduced and the LoD improved, further enhancements in test performance are still possible. For instance, lowering the LoD of specific regions below 0.001% could be achieved by increasing the number of monitored targets, potentially reducing the required input DNA amount, which is clinically advantageous. Additionally, the specificity of hotspot regions could be improved by incorporating advanced noise-to-signal discrimination technologies. We anticipate these improvements will be realized in future iterations.

While the results of this paper demonstrate the analytical performance of the developed diagnostic assay, a limitation is that it has yet to undergo clinical validation. However, we are currently applying this assay in large-scale, prospective clinical studies for colorectal cancer (n = 1,200), as well as in prospective studies for breast and stomach cancer. The data generated from these ongoing studies are expected to provide the necessary clinical validation for the assay.

With a detection limit of 0.001% and an analytical specificity of 99.9%, CancerDetect^TM^ enables clinicians to provide more precise guidance to cancer patients based on their current MRD status and to identify high-risk recurrence groups earlier than traditional clinical methods. Thus, we believe that CancerDetect^TM^ has substantial potential for clinical application.

## Supporting information

S1 Fig The interference test result of bespoke region of CancerDetect^TM.^ The p-value was calculated using Wilcoxon’s test. (A) The observed ctDNA fraction of wild type reference gDNA mixture samples. (B) The observed ctDNA fraction of 0.001% of test. None: The test without additional interference material.
(PDF)

S2 Fig The interference test result of hotspot region of CancerDetect^TM.^ The p-value was calculated using Wilcoxon’s test. (A) The specificity of commercially available 0% reference standard. (B) The sensitivity of commercially available 0.1% reference standard. None: The test without additional interference material.
(PDF)

S1 Table Genes covering clinically actionable targets in CancerDetect^TM^ test. (XLSX)
