# Supplementary figures and images for: Analytical validation of a hybrid-approach combining tumor-informed and tumor-agnostic bespoke ctDNA panel assay for the sensitive detection of minimal residual disease
Source: PLoS One. 2025 Nov 10;20(11):e0334282. doi: 10.1371/journal.pone.0334282 (PMC12599964; doi:10.1371/journal.pone.0334282)

(A)

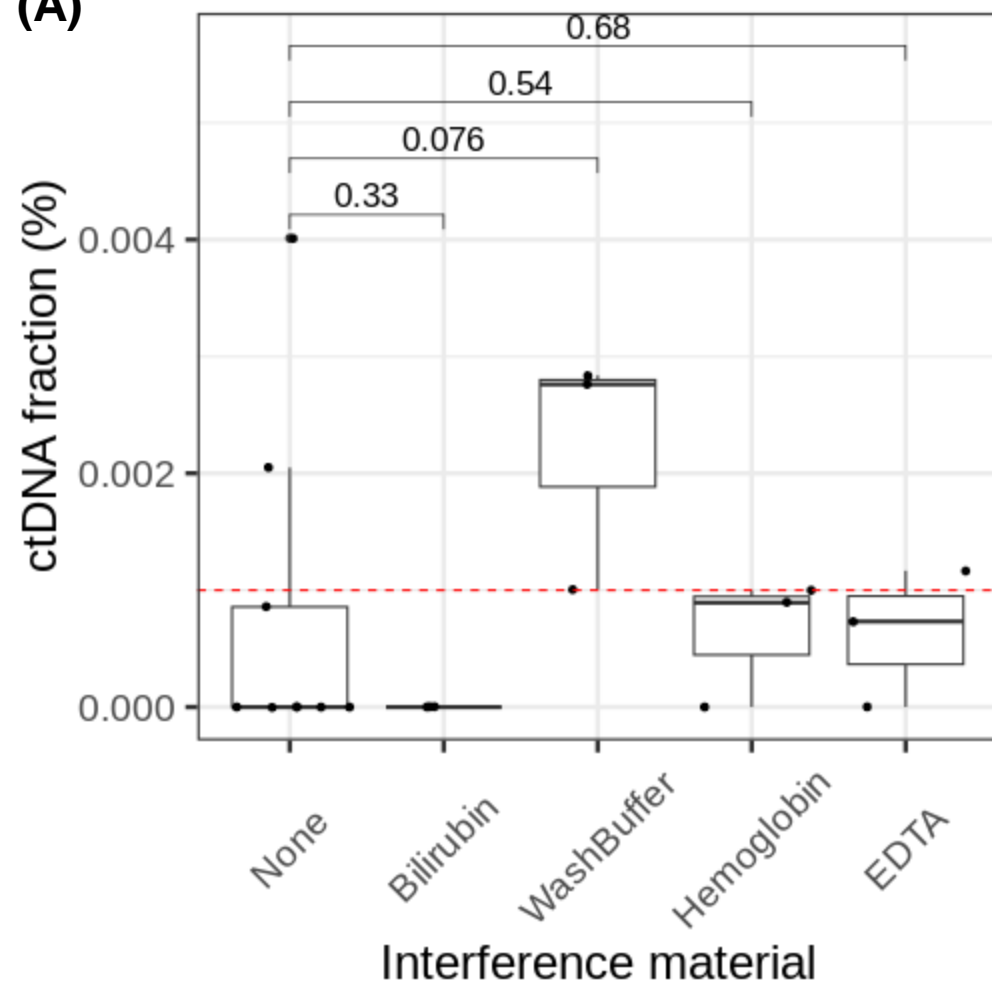

(B)

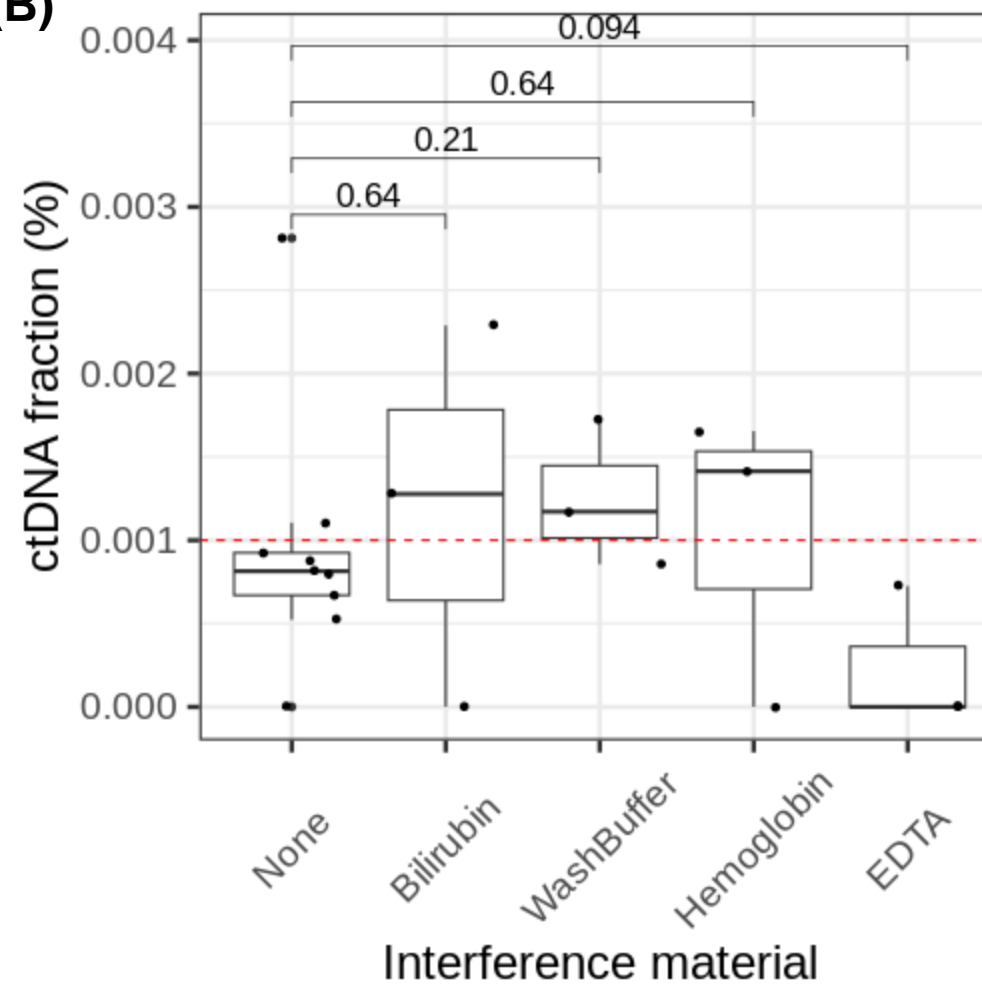

Supplement: S1 Fig — The p-value was calculated using Wilcoxon’s test. (A) The observed ctDNA fraction of wild type reference gDNA mixture samples. (B) The observed ctDNA fraction of 0.001% of test. None: The test without additional interference material. (PDF) [file pone.0334282.s001.pdf]

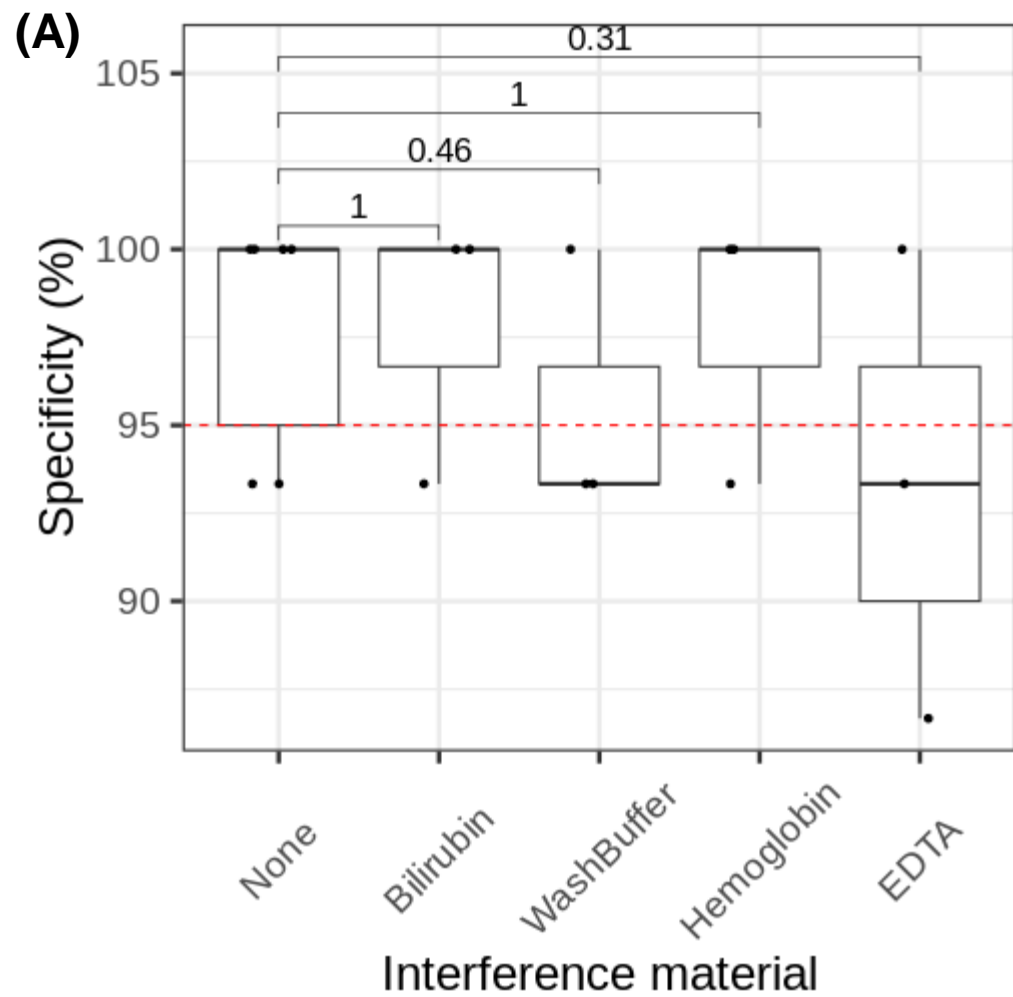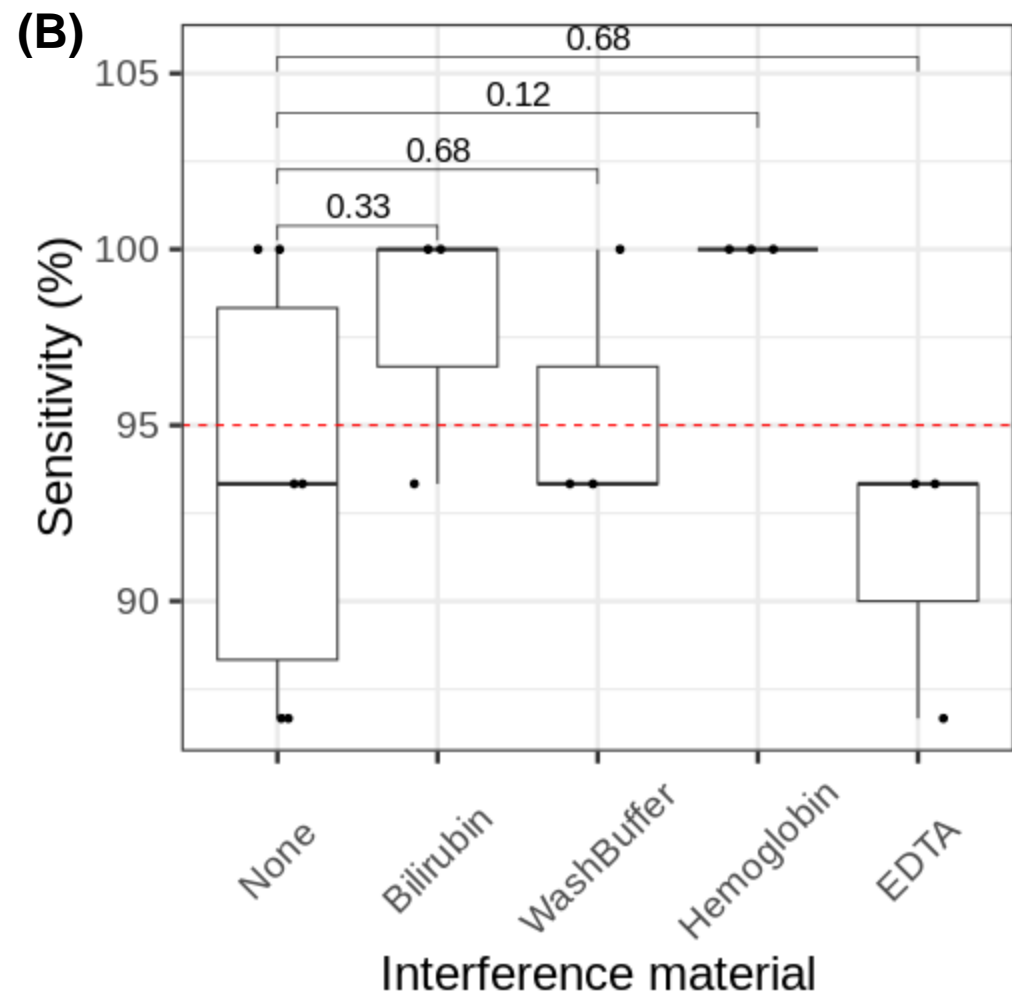

Supplement: S2 Fig — The p-value was calculated using Wilcoxon’s test. (A) The specificity of commercially available 0% reference standard. (B) The sensitivity of commercially available 0.1% reference standard. None: The test without additional interference material. (PDF) [file pone.0334282.s002.pdf]
